# The role of computed tomography in the diagnosis and treatment of calcaneal fractures

**Published:** 2014

**Authors:** F Bica

**Affiliations:** * Orthopaedic Clinic, “Bagdasar–Arseni” Emergency Hospital, Bucharest, Romania

**Keywords:** CT scan, calcaneal fractures, Sanders Classification

## Abstract

The intra articular fractures represent a challenge for the orthopaedic surgeons. Because of the three-articulary surfaces, the calcaneus represents a permanent subject of discussion about the investigation and treatment opportunities.

In a retrospective audit of patients hospitalized in our clinic, I tried to identify the opportunity of the CT scan as a preoperative investigation protocol in calcaneal fractures, the results showing that the CT scan represents a mandatory standard in calcaneal fractures.

## Introduction

Considering the anatomy of the calcaneus, the most important area is the superior articular facet that is divided as mentioned below.

The superior surface is divided in three parts: the anterior third is partly articular (it contains an articular facet for the talus, which it articulates with) and partly non-articular corresponding to the anterior apophysis; the middle third contains the articular facet placed on the sustentaculum tali; the posterior third is formed by the calcaneal tuberosity. From all these zones, the most important is the middle one containing the articular facet for subtalar joint [**1**].

With all these articular facets, the fractures in these areas consider the reduction sequence of the surgery as the main point because of the calcaneus’ role in the foot stability.

The talus sustains the pressure from the leg and spreads it through the contact points of the foot to the ground, with its trabeculae continuing the ones of the tibia, from the level of the plafond and forms two systems, one going to the calcaneal tuberosity and talonavicular joint and the other going to the bones forming the medial arch of the foot. 

The pressure transmitted to the talus via the tibial plafond converges to the lateral and posterior parts of the talus. Therefore, the center of the pressure is projected behind and outside the rotatory axis of the foot, the weight being transmitted to the central axis of the calcaneus, with the talus maintained in its transverse equilibrium position. With its movements, the talus takes the center of pressure of the tibia to the main axis of the calcaneus, an axis that goes through the two weight-bearing tubercles and in the middle of the great apophysis and outside the main axis of the foot. The anatomical feature of the transverse equilibrium of the talocalcaneal joint is the outside projection of the pressure forces, the whole foot arch being rotated to the lateral edge of the foot [**2**].

The Bohler angle description led to the Merle d`Aubigne classification.

In 1951, Merle d`Aubigne divided the fractures as it follows:

**Talus fractures**

-simple which contain the transverse fractures (subtalar fractures, fractures involving the anterior part of the talus and posterior part of the talus) and sagittal fractures involving the sustentaculum tali.

-collapsing fractures which imply the Bohler angle, which is the angle formed by the two lines: one line unites the superior pole of the articular facet with the most superior part of the anterior apophysis and the other line is tangent to the tuberosity. Normally, its values are between 25-40 degrees according to Bohler and 20-40 according to other authors:

Gr I - Bohler angle <30 degrees

Gr II - Bohler angle around zero degrees

G III - Bohler angle reversed

**Extra-articular fractures**

-fractures of the postero-superior angle

-fractures of the calcaneal tuberosity

-fractures of the plantar tuberosities 

-fractures of the great apophysis [**2**]

This classification was heavily used in clinical practice, but the lack of the fracture pattern description led to the necessity of other forms of investigation.

## Materials and Methods

This paper is a prospective study during 13 years (01.01.1997-31.12.2010), conducted in the Orthopaedic Department of “Bagdasar-Arseni” Emergency Hospital, Bucharest. 

It included 250 patients with calcaneal fractures admitted on our ward. 

The following parameters were taken into consideration:

-age

-gender

-fracture type according to Sanders classification

-treatment method

-immediate complications

-late complications

The investigation protocol for all the patients included the standard lateral X-ray (for Bohler angle), Broden view, and coronal and axial CT scan with slices of 3mm.

The three main objectives of the CT scan are the following:

-to determine the number of fragments of the subtalar joint

-to evaluate the relationship between the sustentacular fragment and the rest of the calcaneus

-to evaluate the width loss of the calcaneus

If it can be obtained, the axial and the coronal 3 mm slices are usually enough. If sections in only one plain are obtained, then the slices should be of 1-2mm in dimension. Generally, the evaluation of the articular congruence and the articular facets are better observed when the CT scan slices are perpendicular to the respective area. From the axial and coronal sections, the best view for the subtalar joint is the coronal sections. The calcaneocuboid joint is best seen on the axial view. When it is not possible to obtain the coronal sections, it can be reconstructed by using the axial slices. The CT scan evaluation of both feet can be useful, comparing them and also noting a potential lesion on the other side [**3**].

The evaluation of the CT scan can be eased by knowing the most frequent calcaneal fracture patterns. Most of the intra-articular comminuted fracture patterns are in a sagittal plane or approximate. This fracture line usually involves the subtalar joint. It can have a slightly oblique pattern from the posterolateral region to the anteromedial region of the calcaneus, mostly involving the calcaneocuboid joint. 

There are also other fracture lines at the intersection of the sagittal line, usually in a coronal plane forming a T or a Y fracture pattern.

As a conclusion, the CT scan can really help in the evaluation of the intra-articular fractures of the calcaneus, mostly in the evaluation of the subtalar joint, the calcaneal width and whether it involves the calcaneocuboid joint. CT scan should always come as a plus to standard X-rays because these are also useful in evaluating some fracture features [**4**].

Sanders described a classification of intra-articular fractures based on the coronal and axial CT scan in the widest portion of the posterior area of the calcaneus:

-Type I fractures-are fractures without displacement 

-Type II fractures-are two part fractures of the posterior articular facet 

-Type III fractures-are three part fractures

-Type IV fractures- are four-part fractures or highly comminuted fractures

## Results

**Table 1:** Repartition of cases according to age

20-29	12%
30-39	24%
40-49	33%
50-59	16%
60-69	12%
70-100	3%

**Table 2:** Repartition of cases according to gender

female	8%
male	92%

**Table 3:** Causes of calcaneal fractures

accidents	5%
falls	221%

**Table 4:** Repartition of cases according to Sounders Classification

Tip 1	0%
Tip 2	58%
Tip 3	35%
Tip 4	7%

**Table 5:** Percentage of cases with functional treatment

Functional treatment	4%
Other types of treatment	96%

**Table 6:** Ways of fixing

	brooch	mixed	molded plate	screws
ways of fixing	128	10	95	7

**Table 7:** With/ without complication

Without complication	With complication
218	22

**Table 8:** Late/ immediate complications

late complications	immediate complications
81	159

## Discussion

The Normal Anatomy of the unfractured calcaneus is complex because of the talar joint and cuboid joint. The complicated relationship between them is the basis for complex kinematics subtalar movement, which enables the walking. A good understanding of the anathomopathology of fractures of the calcaneus and their impact is essential for the understanding of the methods of treatment applied for calcaneal fractures.

Letournel proposed the concept of a constant line separation to describe a common bone damage. This constant line separation has a sagittal axis longitudinal trajectory of the posterior facet to the joint or facet of the calcaneus – cuboid or both. The guideline regarding the fractures line is in accordance with the concepts postulated by Palmer and Essex – Lopresti, from their clinical experience and has been confirmed by studies of Hamilton and Bear (1989).

The anterolateral and sagittal line is found in both models.

The computed tomography imaging enhanced the appearance of calcaneus, in particular the anterior region of the posterior talocalcaneal joint. Despite the possibility of correctly describing the anterior extension of the primary fracture line, many authors continued to emphasize the role of the posterior facet. Recent studies have begun to pay an increasing attention to the anterior and medial joints. We believe that both the anterior and posterior subtalar joint should be considered during treatment, especially if surgical.

The surgical treatment of the calcaneal fracture surface restoration joint provides a good result, a poor joint alignment while being associated with posttraumatic joint degeneration.

The aim of the surgical treatment in the articular fractures and articular calcaneal fractures in particular is to reduce the congruence of articular surfaces perfectly. The cases presented showed serious challenges of the articular fracture treatment calcaneus, due to the limited visibility attempts to reduce the articular fragments, often being the lowest rung or inclination deviations invariably leading to subtalar arthrosis and thus failing reconstruction.

Thus, all the 18 cases of calcaneal fractures, Saunders IV, were solved by primary surgical reconstruction – arthrodesis. To these cases, 5 other in which the surgeon Saunders III appreciated timely reconstruction – arthrodesis, were added. Wagner experiments indicated that the pressure level of the previous facet joint is 50% greater than pressure in the posterior facet joint, this pressure being significantly modified by a simple shift of 2 mm of the talar neck, so that, it can be concluded that the reduction of the articular surfaces prior failure can produce the same effects .

## Conclusions

We consider that the pre-operative calcaneal CT reconstruction scan is mandatory in the pre-operative planning of the calcaneal fractures with articular collapse because of the added information it provides considering the secondary fracture lines involving the anterior and the main articular facets of the calcaneus. 

From our experience, only Sanders fracture Type II and Type III (which corresponds only to a part of the Type II Bohler fractures) are amenable to the surgical reconstruction and osteosynthesis, the main objectives being the restoration of the lateral height and the anatomical angles of the calcaneus. In all Sanders fractures Type II and Type III, the surgery is indicated per-primam to accomplish the reconstruction and osteosynthesis.

In all Sanders Type IV fracture patterns, the main indication is arthrodesis-reconstruction.

The functional outcome of every type of surgical intervention is dependent on the right indication for surgery, pre-operative planning, and the skills of the surgeon to understand and reconstruct the calcaneal anatomy, to restore the lateral height, the calcaneal width and the congruity of the articular facets. 

The Bohler classification does not represent a major and decisive factor in the preoperative planning of the calcaneal fractures, mostly having a didactic value.
